# For the Prevention of Mistakes in the Handling of Medicines

**Published:** 1896-06

**Authors:** 


For the Prevention of Mistakes in the Handling of Medicines. According to an ordinance of the Ministry of the Interior of Oct. 2, 1895, for preventing the mistakes in the handling of medicine, powerful drugs must be kept and dispensed in bottles of a *particular* shape which shall be provided with a durable label. The use of paper labels for such medicines is strictly forbidden. The editor of the Austrian *Sanitätswesen* remarks: Paper labels are superscriptions upon paper which are merely fastened to the bottle by means of paste. If the writing on the paper by some corresponding means of stamping or coating over is fixed inseparably to the glass wall of the bottle, and so that it cannot become blurred or blotted out, it is no longer a question of mere paper labels. According to this interpretation paper signatures, if they are coated over with collodium and lacquered, are admissible. The ordinance concludes with the regulation that the measure be brought to the knowledge of the associated physicians and the association of apothecaries in order that a strict observance of the law may be attained. Since the interpretation in regard to the paper signatures may be considered as official, some agreement as to the shape of the different bottles to be used seems to be all that is now needed.—*Ibid.*

